# Modern Methods of Pre-Treatment of Plant Material for the Extraction of Bioactive Compounds

**DOI:** 10.3390/molecules27030730

**Published:** 2022-01-23

**Authors:** Aneta Krakowska-Sieprawska, Anna Kiełbasa, Katarzyna Rafińska, Magdalena Ligor, Bogusław Buszewski

**Affiliations:** 1 Department of Environmental Chemistry and Bioanalytics, Faculty of Chemistry, Nicolaus Copernicus University, Gagarina 7 St., PL-87100 Torun, Poland; akra@doktorant.umk.pl (A.K.-S.); kielbasam@umk.pl (A.K.); katraf@umk.pl (K.R.); Magdalena.Ligor@umk.pl (M.L.); 2 Interdisciplinary Centre of Modern Technologies, Nicolaus Copernicus University, Wileńska 4 St., PL-87100 Torun, Poland

**Keywords:** sample preparation, plant material, drying, freeze-drying, convection drying, microwave vacuum drying, enzymatic processes, fermentation

## Abstract

In this review, recent advances in the methods of pre-treatment of plant material for the extraction of secondary metabolites with high biological activity are presented. The correct preparation of the material for extraction is as important as the selection of the extraction method. This step should prevent the degradation of bioactive compounds as well as the development of fungi and bacteria. Currently, the methods of preparation are expected to modify the particles of the plant material in such a way that will contribute to the release of bioactive compounds loosely bonded to cell wall polymers. This review presents a wide range of methods of preparing plant material, including drying, freeze-drying, convection drying, microwave vacuum drying, enzymatic processes, and fermentation. The influence of the particular methods on the structure of plant material particles, the level of preserved bioactive compounds, and the possibility of their release during the extraction were highlighted. The plant material pre-treatment techniques used were discussed with respect to the amount of compounds released during extraction as well their application in various industries interested in products with a high content of biologically active compounds, such as the pharmaceutical, cosmetics, and food industries.

## 1. Introduction

The step of sample preparation for extraction and chromatographic analysis has attracted the attention of researchers, analysts, and companies producing analytical equipment for almost two decades. This interest shows the awareness of the importance and impact of this stage of the analytical procedure on the quality of the results obtained. Moreover, it results from the general tendency to use faster and more effective methods which are ideally more environmentally friendly [1].

Proper preparation of material for research and many branches of industry, such as pharmaceutical, cosmetics, and food, is fundamental in extracting and analyzing complex natural materials. Proper sample preparation can increase the extraction efficiency of biologically active compounds. These preparations include weighing, volume measurement, mixing, diluting, heating, cooling, fractionation, purification, and preservation. Weighing is the first stage responsible for the final result of the analysis. The basic task is to ensure the right number of samples are taken for analysis. The volume measurement is used for liquid and gaseous samples. Mixing is one of the steps in the initial sample preparation. It must ensure the homogeneity of the sample and is also used when diluting samples. The heating of the sample is necessary when dissolving or removing water from the material, and lowering the temperature is used for preserving the sample and, e.g., during freeze-drying. Sample purification and fractionation are based on sieving, filtration, sedimentation, centrifugation, foam flotation, membrane filtering, distillation, evaporation, and crystallization. The purification process is often connected with the extraction, i.e., the isolation of analytes from the sample. Therefore, we can also distinguish the technique of solvent extraction (e.g., liquid-liquid), solid-phase extraction, or preparative chromatography. Preservation is intended to protect the sample during processing against a change in analytes’ content as their loss during physical or chemical processing changes the nature of the matrix. All of these activities affect the final yield of extraction [2,3].

The distinguishing feature of plant material, apart from its variability and the characteristic structured cell architecture, is the abundance of compounds with different physicochemical properties which occur in a wide range of concentrations. Plant material is a very complex matrix composed of a wide variety of plant metabolites, substances with a wide range of polarity. These include waxes, terpenoids, lipids, phenolic compounds, low-polar alkaloids, polar glycosides, polar alkaloids, saccharides, peptides, and proteins. Bioactive compounds are primarily phenolic compounds (including flavonoids), saponins, cyclitols, etc. A good method of preparing a plant sample should be expected to, regardless of the location of a given compound in the matrix, its type, or the presence of other components (e.g., lipids), quickly and thoroughly isolate the volatile and non-volatile, the polar and non-polar compounds, and be sensitive and resistant, e.g., to high temperature. As a result of a well-chosen method of preliminary sample preparation, phytoactive ingredients become more available and, at the same time, easier to extract. Considering the area of use of raw or pre-processed plant material and the importance of the analysis being carried out, the lack of influence of the method used to change the qualitative and quantitative composition of the tested plant is a particularly desirable feature of the method of preparing a plant sample [3,4,5].

The preparation of a sample of plant material for analysis involves several important steps. The first step is pre-washing, drying or freeze-drying the plant material, and grinding for homogenization. The following steps are extraction and qualitative and quantitative determination [1,2,6].

When planning the pre-treatment processes of plant material, several aspects characteristic for a given type of raw material should be taken into account, which, depending on the needs, may be a disadvantage or advantage. In the course of raw material processing, its components usually undergo irreversible chemical changes such as oxidation, hydrolysis, or the condensation of components (observed, for example, in the case of polyphenols which can form strong and potentially irreversible adducts with the cell wall material). Because the plant cell wall is a highly complex matrix, proper preparation is crucial for obtaining a high isolation efficiency of biologically active compounds. Additionally, regardless of whether the raw material was initially pre-treated, its quality will be influenced by many other factors including the way the plant was grown, the place where it was grown, weather conditions, the method of drying the biomass, temperature, the final water content of the raw material, and its storage temperature. All of these factors will influence the final chemical composition of the biomass [7,8]. Importantly, the water content varies greatly. Factors that influence it are plant species, its development stage, and growing or storage conditions. The pre-treatment processes assist in the destruction of cell walls by enabling and/or facilitating the release of active ingredients. Depending on what biologically active compounds we want to release and what plant material we are dealing with, we can subject the material to the process of drying, freeze-drying, fermentation, or enzymatic hydrolysis. Preparation of a plant sample can be problematic when analytes are unstable compounds that can be lost or undergo chemical modification during preparation [1,2,9,10,11,12].

Bioactive substances isolated from plant material can have antioxidant, antimutagenic, anticancer, antiemetic, antifungal, antibacterial, and many other properties. They are used to treat and relieve symptoms of many diseases. They can be used in the food, cosmetic, pharmaceutical, and agricultural industries as well as many other sectors of the economy [1,10,11,12].

In this review, the pre-treatment techniques of plant material were described. However, method selection is very important for both analytical research at the laboratory level as well as for the industrial level where the main aim is to extract as much bioactive compounds as possible. We focused on conventional methods such as convection drying, microwave vacuum drying, and freeze-drying, as well as paying attention to the increasingly used modern methods: fermentation and enzymatic hydrolysis. We also presented the influence of the discussed sample preparation methods on the content of bioactive compounds in plant material. So far, the techniques mentioned that are currently used in the extraction industry are limited to drying or convection drying. Most of the presented works refer to a laboratory scale but their effectiveness should lead to the transfer of the presented ideas to an industrial scale.

## 2. Drying the Plant Material

Research on medicinal plants begins with pre-extraction, i.e., the initial preparation of the raw material, which is an important stage in processing plant materials. This stage should prepare samples in such a way as to limit the loss of the bioactive ingredients contained in the plants. Preliminary raw material preparation steps such as grinding and drying can also influence the phytochemical content of the final extracts. Many authors, especially those using plants used in ethnomedicine, prepare fresh tissue extracts. However, dried plants are used more often [13].

The drying process is primarily aimed at inhibiting metabolic processes which result in stopping changes in the plant’s chemical composition. This occurs by removing water from the plant material, which is necessary for plant enzymes to function properly. The lack of water and a high drying temperature contribute to the inhibition of enzymes that can break down the active ingredients. Proper drying also reduces the number of microorganisms in the final product. It significantly reduces the weight and volume of plant material, reducing the costs of packaging, transport, and storage of plant substances [14,15].

The drying of the plant material may take place under natural or artificial conditions. The former includes drying in the open air or semi-open rooms, where free air circulation is used, but there is no direct sunlight. Drying carried out in natural conditions is the oldest method of preserving raw material. Nowadays, this method is abandoned because the process is long and does not allow for the adjustment of drying parameters, which means that the quality of the obtained dried material is low. Drying in artificial conditions includes ventilated rooms with various heating methods, where we can set the temperature range and air pressure [15,16,17].

The method of drying the plant material largely depends on the active ingredients it contains. If the plant material is a source of flavonoid compounds, drying at high temperatures can be used (even up to 100 °C and 180 °C) [16,18]. Applying a high drying temperature commonly leads to the loss of volatile compound content. Additionally, high drying temperatures could promote the degradation of heat-labile compounds in the essential oil [17].

Drying is of great importance primarily in the food industry. The quality of the products guarantees that their features will remain unchanged over a long period of time with a high level of consumer acceptance. Effective and thorough drying methods are required to guarantee the high quality of the products obtained. Drying on a laboratory scale can be carried out in laboratory dryers, and on an industrial scale in a vacuum chamber, tunnel, spray drum, tower, and shaft dryers [14,17].

### 2.1. Convection Drying

**Figure 1:**
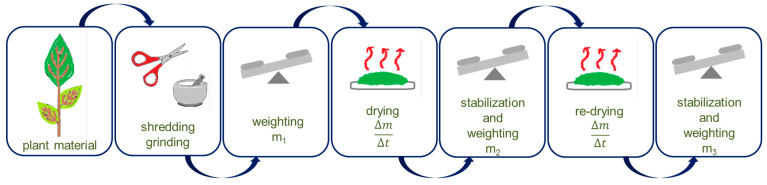
The steps of convection drying (m_1_—weight of the sample before drying; m_2_, m_3_—sample weight after successive drying; $\frac{\Delta m}{\Delta t}$—change in mass of the sample during drying).

The most common method for plant material preparation is convection drying. Convection drying is one in which a stream of drying agent (dry gas, most often air) flows around the plant material bringing heat and removing moisture (Figure 1). The drying process is a process of simultaneous heat and mass exchange as well as an ongoing phase transformation (conversion of water into steam). The process of drying a solid is non-stationary, i.e., both the temperature of the dried material and its water content change continuously during the process. In the process of convection drying, the phenomena of external heat and mass exchange occur between the surface of the material and the gaseous environment and the internal heat and mass exchange inside the solid [19]. The greatest advantage of this method is the possibility of obtaining a relatively cheap product but at a significantly reduced quality. Namely, during convection drying, several physical and physicochemical changes of the material occur. The most important parameter of convection drying is the temperature of the drying agent, which is usually air. With increasing air temperature, the drying rate increases [20,21].

Drying conditions and the plant species affect the content of bioactive substances. Both the temperature and the drying time are of great importance for bioactive compounds, which indicates that each description of the methodology should precisely define the conditions of the experiment. For example, the vitamin C content of dried stevia (*Stevia rebaudiana*) leaves decreased with increasing drying temperature, although it remained relatively high [18].

Akbudak et al. [22] assessed the effect of the drying method on the vitamin C content in parsley leaves. Taking into account convection drying, it was observed that the degree of vitamin reduction increased with the extension of the drying time, which ranged from 20 min to 116 min for temperatures from 125 °C to 50 °C. Guine et al. [23] conducted qualitative tests of pumpkin dried with the convection method using various temperatures from 30 °C to 70 °C. The authors compared the content of, among others, proteins, lipids, and sugars in fresh and dried pumpkins. It showed that the smallest changes in chemical composition took place when the lowest temperature (30 °C) was applied, but the results were not much worse when when using the highest temperature. The research confirmed that the factors influencing the quality of the dried material are the temperature and the drying time, which was 8 h for 30 °C and only 2 h for 70 °C [23]. In the case of examining the chemical properties of pears dried using the convection method, a significant effect of temperature and time on the content of vitamin C and phenolic compounds was demonstrated [24]. In addition, it was observed that the samples of stevia leaves obtained after 10 h of drying at 30 °C had the lowest total phenol content, while the samples obtained after 2 h of drying at 70 °C had a slightly higher content of these compounds. The antioxidant activity measured by the ORAC method (oxygen radical absorbance capacity) also showed the highest value at 40 °C, indicating favorable drying conditions [25]. After the drying process, a decrease in the content of phenolic compounds and antioxidants was also found in garlic and tomatoes [26].

Leng et al. [27] compared the total content of phenolic compounds and the antioxidant capacity of fresh tamarind (*Tamarindus indica* L.) leaves dried at 60 °C for 3 h or dried at 180 °C for 10 min. The total antioxidant capacity and total phenolic compound content in the extract were highest in the case of leaves dried at 180 °C. Similarly, when comparing the fresh and dried leaves of *Moringa oleiefera*, it was found that drying did not affect the total phenolic compound content but increased the concentration of flavonoids [28].

Air drying usually takes a long time—from several days to several months, depending on the type of plant parts being dried (e.g., leaves or seeds). Plant samples, usually leaves and stems, are left in a shaded, airy place at ambient temperature. During drying, plants can become contaminated and are exposed to unstable temperature conditions. Temperature-controlled oven drying can be useful in drying plants rich in essential oils. The highest content of essential oil in lemon verbena (*Lippia citriodora*) was obtained by drying in an oven at 30 °C [29]. However, it was shown that increasing the heating temperature in the oven from 30 °C to 50 °C lowered the content of essential oils in the leaves of *Laurus nobilis* L. and *Artemisia annua* [30,31].

The course of the convection drying process depends largely on the degree of fragmentation of the raw material which increases the speed and efficiency of extraction. However, in the descriptions of the methodology of extract preparation, the technical parameters of this stage are usually omitted. The degree of fragmentation of the material affects the extraction efficiency, i.e., the fineness and small particle size increase the surface contact between the samples and the extraction solvents. The powder step and sieving increase the homogeneity of the sample and the reproducibility of the extraction [32]. Gião et al. [32] showed that the total antioxidant content in aqueous extracts of medicinal plants depended on particle size and extraction time. The effect of the extraction time depended on the plant species, but approx. 5 min was sufficient to ensure an acceptable degree, and the smaller particle size increased the antioxidants contained in the extract. Therefore, the authors recommend the use of ground material with a particle diameter of 0.2 mm. Particle size is significant when enzyme-assisted extraction is used. Additionally, during the supercritical extraction of essential oils from parsley seeds, the particle size (293 to 495 nm) was one of the important parameters influencing the course of the process [33].

Convection drying is most often used on an industrial scale. The disadvantage of this method is combination of the long drying time and high temperature which can, in some cases, lead to the degradation of valuable nutrients and aromatics [34].

### 2.2. Freeze-Drying (Lyophilization)

**Figure 2:**
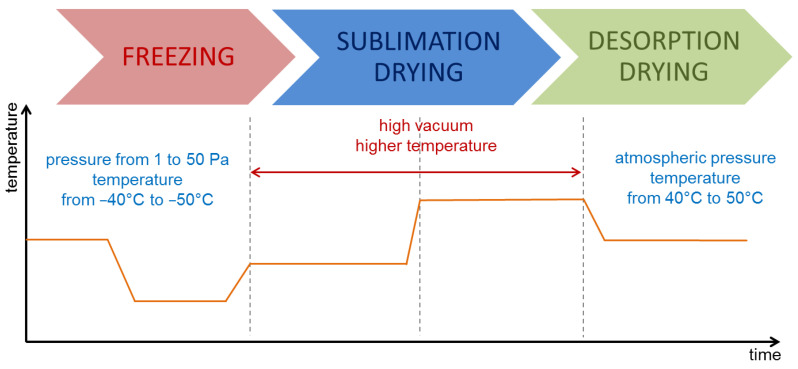
Diagram of the freeze-drying process.

Freeze-drying is another method of product drying where the main idea is to remove water from the frozen material using the ice sublimation process. This process is called lyophilization or molecular drying. The quality of the obtained dried material is much higher than in other drying methods. Generally, this process removes water from a material using sublimation, i.e., a solid-to-gas transition bypassing the liquid phase. It is carried out in negative temperature conditions and significantly reduced pressure (1–50 Pa). The first and crucial step in this process is freezing at a temperature of −40 °C to −50 °C and is relatively short-lived to prevent the formation of large ice crystals. The next step is the freeze-drying using a combination of vacuum and temperature. This procedure allows for the removal of up to 95% of the water and can take up to 2 days. In this process, moisture does not condense into a liquid but condenses in a volatile state. The last stage is re-drying, called desorption, which occurs at 40–50 °C. Its purpose is to eliminate the strongly bound (chemically) water that has not been frozen and remains in the dried material. As a result, only 1–2% of water remains in the freeze-dried material [35,36]. The stages of the freeze-drying process are shown schematically in Figure 2.

There are significant differences between freeze-drying and drying that make freeze-drying the better method of sample preparation. Standard drying methods may cause chemical or physical changes to the product due to high temperatures. In the case of delicate plant extracts, this can significantly affect the quality of the end product. The differences mentioned above include: the method of removing water, the amount of water removed (freeze-drying removes as much as 98% of the water from samples, while conventional drying removes 70–80%), the shelf life of the product obtained (freeze-drying of fruits and vegetables allows them to be stored for 20–30 years, while in the case of drying, their shelf life varies from 1 to 5 years), and the nutritional value (freeze-dried products have much more vitamins and nutritional value than those dried traditionally) [35,36,37].

**Figure 3:**
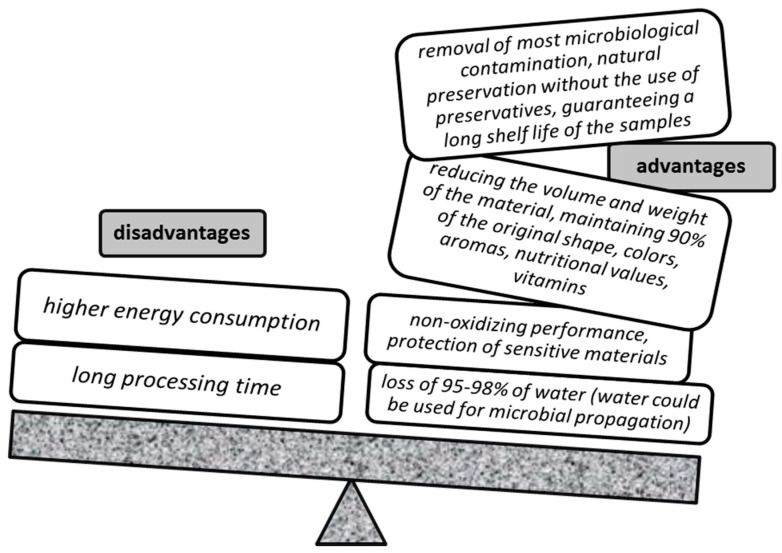
Advantages and disadvantages of the freeze-drying process.

Freeze-drying has many advantages, but there are also some disadvantages [38] (Figure 3).

The freeze-drying process is mainly used for delicate, temperature-sensitive plant materials on both a laboratory and industrial scale. The process of freeze-drying plant samples ensures high efficiency in removing water, while at the same time retaining bioactive ingredients, including antioxidant compounds. Due to its advantages, the freeze-drying process is also widely used for ensuring the durability and stability of biological materials. It protects against the multiplication of microorganisms as well as the decomposition of biological substances related to the action of microorganisms. It has found wide application in the pharmaceutical, biotechnology, and food industries [38,39].

The freeze-drying process is also used to prepare plant and food samples for the determination of biologically active compounds. It is used to stabilize, enrich, and/or extend the life of the plant material without destroying its chemical structure. In research by Elshaafi et al. [40], the content of TPC and TFC in *Ficus carica* increased in following conditions of plant material preparation: (i) drying the samples at 40 °C, (ii) at 60 °C, and (iii) then as a result of freeze-drying. The content of phenolic bioactive compounds in Asian pear powder [41] turned out to be lower for the freeze-dried samples than those subjected to hot air drying. On the other hand, the antioxidant activity of the bound phenolic compounds was higher in the samples after lyophilization than those subjected to conventional drying. In the research by Pérez-Gregorio et al. [42], an increase in the content of flavonoids and flavonols in the extracts from freeze-dried red onions was noted. In the research on the influence of drying and freeze-drying on the content of bioactive compounds in Romanian grape varieties, Oprica et al. [39] noticed that the content of flavonoids in the skin was the highest in fresh and then freeze-dried material. The lowest levels of these compounds were found in the oven-dried sample. Each freeze-dried pulp sample was characterized by a higher content of flavonoids than in the fresh sample. In the case of fresh seeds, a higher content of flavonoids was found compared to those subjected to freeze-drying. Sun et al. [43] found that the antioxidant capacity of different citrus species dried by freeze-drying was higher compared to those dried by hot air or sun drying. Other results are presented in an article by Papoutsis [44], who demonstrated that the samples dried by hot air or vacuum had a higher antioxidant capacity compared to those dried by freeze-drying. Only for neohesperidin (a flavanone glycoside) was a higher content achieved in lyophilized samples, as drying at higher temperatures caused its content to drop.

Scanning electron microscopy analysis of *Lepidium sativum* showed that the mass transfer is probably higher for dried material than for freeze-dried. The reason is due to the additional damaged structure of dried material which is related to its larger area. In freeze-dried material, most of the cells are not damaged hence the mass transfer, which is the rate-controlling step during extraction, is limited [5].

In the food and pharmaceutical industries, freeze-drying is used on a large scale as the final preparation and protection of the product. This process is applied for the preparation of coffee, tea, and crispy fruits and vegetables in the food industry. In the pharmaceutical industry, it is used for the preservation of microorganisms, enzymes, and pharmaceuticals. Therefore, it is technically possible to prepare a large amount of plant material by means of freeze-drying and then carry out the extraction process on an industrial scale.

### 2.3. Microwave-Vacuum Drying (VM)

**Figure 4:**
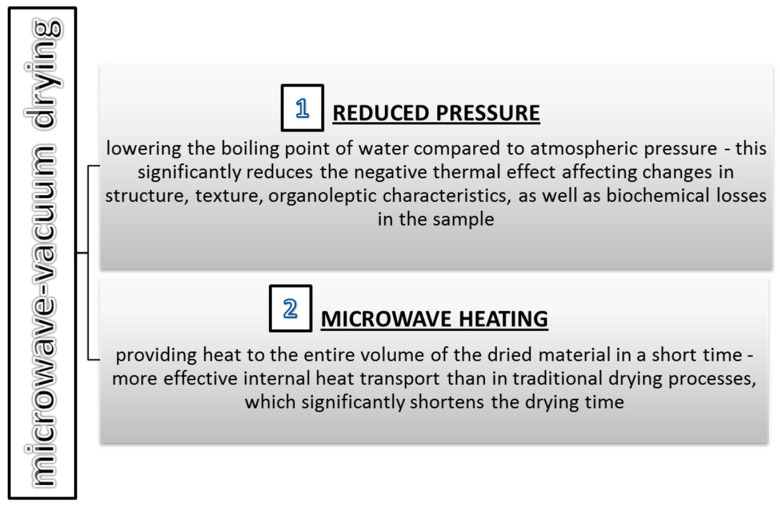
Two important parameters in microwave-vacuum drying.

Microwave-vacuum drying is a modern method that combines the advantages of microwave and vacuum drying [45]. This method can provide a product with excellent properties. This is related to two important issues presented in Figure 4. With appropriate selection and proper control of the microwave power and the pressure range, the microwave-vacuum method enables the rapid removal of water from plant material at a moderate sample temperature with limited contact with oxygen. Microwave drying involves the penetration of a very high-frequency electromagnetic field (300 MHz–30 GHz) into the interior of the dried material. The use of microwaves for drying provides energy throughout the entire volume of the sample which allows for a shorter drying time and thus obtains a high-quality product [46]. In addition, the use of a vacuum allows for an even greater reduction in drying time, limiting the contact of the raw material with air, and lowering the temperature of the material during drying [47].

During drying with the use of microwave heating, it is necessary to select the drying parameters properly. Inadequate process control may lead to a deterioration of the product quality. The use of a too high microwave power may lead to a rapid increase in temperature and, consequently, to the burning of the dried material. Appropriate shaping of the temperature distribution inside the dried material should significantly reduce or even eliminate local overheating of the material. To obtain a final product of the highest quality, it is necessary to optimize the drying process. Inadequate conduct of the process may lead to a deterioration of the quality of the final product. The optimal selection of process parameters will also affect the economic aspect of the entire process [48,49,50].

The basic parameter of VM drying is microwave power, which is often given as power per gram of fresh material (unit power). The level of the applied unit power depends on the type of plant raw material and in laboratory tests it is 0.8–3.0 W g^−1^ of sweet potatoes [51] or 8.0–11.2 W g^−1^ of mint [52]. The increase in microwave power leads to a reduction in drying time. Lowering the pressure in the drying chamber reduces the boiling point of water and increases the pressure gradient between the inside of the dried material and its surface [45,52,53]. Motavali et al. [54] determined the effect of power and pressure on the kinetics of cherry drying using the VM method. The tests used pressures ranging from 20 kPa to 80 kPa. As expected, the drying time was shortened with the increase in microwave power and the decrease in pressure.

Loss of vitamins is a disadvantageous phenomenon during drying. Water-soluble vitamins are susceptible to elevated temperatures, while fat-soluble vitamins are generally less degraded due to oxidation during drying. The consequence of removing water from the fruit is a reduction in the content of water-soluble vitamins, especially vitamin C and fat-soluble vitamins, which reduces the nutritional and sensory value of the fruit. Ascorbic acid is also a substrate of the browning reaction, which is particularly important when drying raw materials rich in this compound [55].

Microwaves, by heating the product in its entire volume, significantly reduce the drying time and therefore it is possible to reduce the loss of thermolabile vitamins. The content of vitamin C in dried apricots obtained using a microwave dryer was higher than in infrared dried apricots [56]. On the other hand, additional pressure reduction during microwave drying may reduce vitamin oxidation, which was observed in microwave-vacuum-dried carrot slices, characterized by a higher content of β-carotene and vitamin C [57].

The applied microwave power also differentiates the degree of vitamin degradation. Increasing the power allowed for the preservation of more ascorbic acid in spinach [58] and nettle [59]. As reported by Alibas et al. [59], at a microwave power in the range of 500–1000 W, the content of ascorbic acid in dried spinach decreased only by about 15%, and at the power of 90–350 W—by about a half, which resulted from the longer process time.

Wojdyło et al. [60] used the power of microwaves to dry strawberries and determined a few basic parameters in their research. The plant material was dried using microwaves using 240, 360, and 480 W powers. The samples vacuum-dried in the microwave with the 240 W power had a higher level of vitamin C, anthocyanins, phenolic compounds, and antioxidant activity compared to the two consecutive strength levels. These were high-quality products. Additionally, it showed that vacuum-microwave drying protects phenolic components and ascorbic acid-sensitive to temperature and oxygen.

Zielińska and Michalska [61] described the dependence between the content of polyphenols, anthocyanins, and antioxidant capacity and the microwave-assisted drying of blueberries. It turned out that TCP and antioxidant activity decreased after drying with microwave vacuum drying compared to hot air convective drying. The opposite is the case with anthocyanins. Their content in the extract is higher after vacuum microwave drying than with hot air. In other research [62], the total phenolic content, expressed in mg GAE/100 g of dry weight, was highest in rosemary leaves subjected to vacuum microwave drying using 360 W microwave power. The same conclusions can be drawn for the antioxidant activity.

## 3. Fermentation as Modern Sample Preparation Method

Fermentation is a biochemical reaction of the anaerobic decomposition of organic compounds with the participation of enzymes produced by microorganisms. It relies on transforming complex substrates into simple compounds by microorganisms such as bacteria, yeast, and fungi. As a result of this process, secondary plant metabolites are released. Fermentation gives the product a characteristically different taste, aroma, and texture. The nutritional value of such a product also changes. We can distinguish among mushroom, alkaline, and lactic acid fermentation. In industry, submerged or solid-state fermentation (SSF) processes are used [63,64].

**Figure 5:**
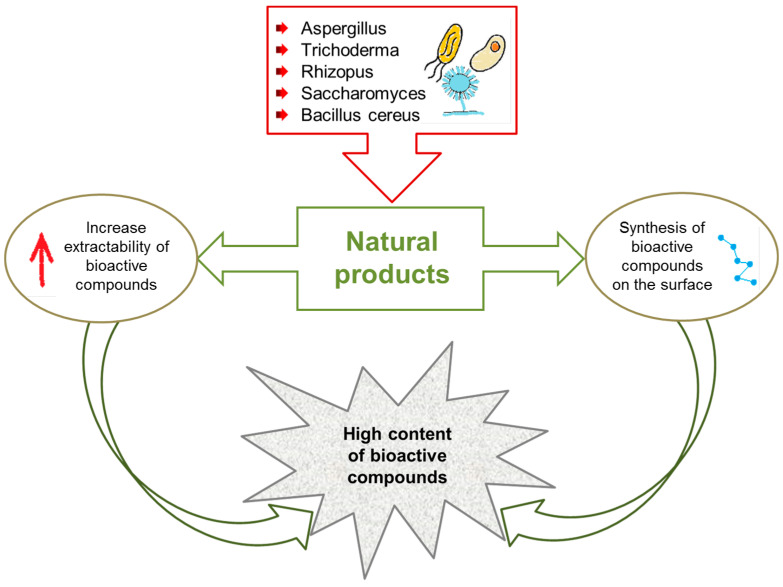
Schematic of an SSF process.

The substrates used in SSF can be divided according to the composition, chemical nature, particle size, surface, mechanical properties, and water holding capacity. The aforementioned parameters influence the efficiency of the conducted process, and they can be used to optimize fermentation [64]. The scheme of SSF is presented in Figure 5.

**Figure 6:**
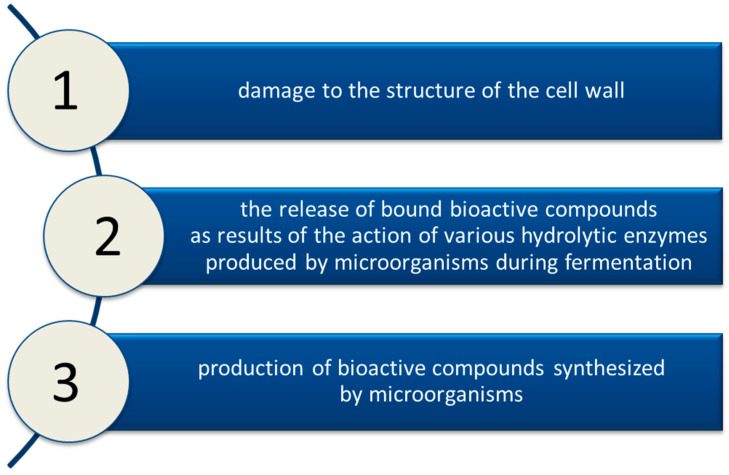
Reasons for using SSF as a method of sample preparation for extraction.

Solid medium fermentation can be divided into several steps. An adequately prepared material (after grinding or fragmentation) is inoculated with microorganisms that will grow on the surface of the material. As a result of the action of the enzymes produced, the hydrolysis of polysaccharides and proteins takes place. This is followed by the biosynthesis of some bioactive compounds and the release of the associated analytes. The final step is the purification (e.g., precipitation, dialysis, column chromatography, and preparative TLC) and determination of the final product. The microorganisms used are fungi, yeasts, and bacteria such as *Bacillus subtilis*, *Lactobacillus delbrueckii*, or *Saccharomyces cerevisiae*. Process optimization is based on the appropriate selection of microorganisms, their substrate, solid-state properties, proper humidity, and temperature, as well as extraction and purification techniques. SSF can be used to produce enzymes (pectinases, cellulases, and amylases), biopolymers (exopolysaccharides, polyhydroxyalkanoates, and dextran), and biosurfactants. The innovative use of SSF is based on the use of fermentation as a method of sample preparation for the extraction or determination of bioactive compounds (Figure 6). The enzymes produced by the applied microorganisms affect the cellular structure of complex substrates and then release bioactive compounds associated with the solid matrix, thus increasing the efficiency of their extraction [64,65].

**Figure 7:**
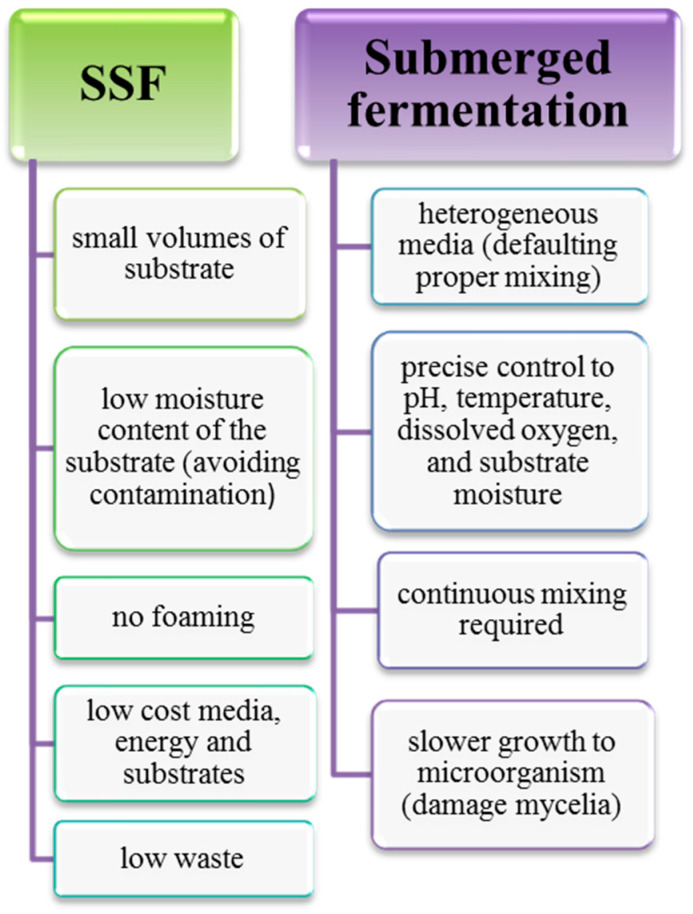
Differences between solid-state and submerged fermentation.

The second type of fermentation is submerged fermentation (SmF). In the submerged fermentation process, liquid substrates are used and microorganisms (as sources of enzymes) and other reactive compounds are immersed in the liquid. Microorganisms grow in the liquid medium and bioactive compounds produced intracellularly are secreted into the fermentation broth after a rupture step. Such fermentation can be carried out continuously or batch-fed. The key parameters in the fermentation process are temperature, pH, oxygen, and carbon dioxide levels. Submerged fermentation is mainly used for the extraction of secondary metabolites that must be used in liquid form. The submerged fermentation technology is characterized by a short time, easy process control, low cost, high efficiency, and simple product purification. Compared to SSF, submerged fermentation generates more wastewater, the concentration of the products is lower, and the properties of the product obtained are inferior [65,66]. The most important differences between the two fermentation methods are shown in Figure 7.

The purpose of fermentation is to partially hydrolyze the polymers of the cell walls, thus releasing the bioactive compounds bound in the cell walls. Before selecting the extraction technique, several key issues should be considered, such as the nature of the matrix, the type of the biomolecules of interest, the scaling of the process, the relationship between production costs, and the economic efficiency of the final product. Solid-state fermentation is a flexible new alternative to the traditional fermentation process (SmF). On the other hand, solid fermentation is constantly being improved due to the desire for greater automation and optimization of the process [65,66]. The development of fermentation techniques makes it possible to produce bioactive compounds at an industrial level (Table 1).

Ajila et al. [78] used solid-state fermentation as a sample preparation before extracting the polyphenolic compounds from apple pomace. For this purpose, crust fungi *Phanerocheate chrysosporium* was applied. The fermentation was carried out at a temperature of 37 °C for 14 days. Next, the bioactive compounds were extracted by ultrasonication and microwave-assisted extraction methods. The obtained results were compared with the content of phenolic compounds for non-fermented apple pomace. The results clearly showed that solid-state fermentation contributed to an increase in the content of polyphenols (4.6 to 16.12 mg GAE/g dry weight) as well as antioxidant activity (range from 12.24 to 23.42 µg TEAC/g dry weight). *Aspergillus niger* and *Rhizopus oligosporus* were used for solid-state fermentation of plum pomaces and brandy distillery wastes in research by Dulf et al. [84]. After liquid-liquid ultrasound-assisted extraction and determination using HPLC-DAD-MS, the amounts of the polyphenols and flavonoids were higher in the extracts after fermentation. Moreover, antioxidant activity was enhanced. Interestingly, fermentation also influenced the quantity and quality of the extracted fatty acids from the PUFA family. Similar results were obtained in research by the same author with white grape pomace [86]. The sample was treated with two fungi: *Actinomucor elegans* and *Umbelopsis isabellina*. After fermentation, lipids, and carotenoids were extracted using liquid-liquid techniques with the corresponding solvents. Determination was carried out by GC/MS or HPLC-DAD-MS. An increase in the content of phenolic compounds (antioxidant activity), lipids, and carotenoids were achieved. Similar conclusions regarding the content of polyphenols (shikimic acid, chlorogenic acid, vanillic acid, rutin, sinapic acid, and luteolin) and antioxidant activity were presented in studies by Xiao [68,74]. In the first case, a mung bean sample was fermented with *Cordyceps militaris* SN-18. In another article, soybeans were processed by solid-state fermentation with *Rhizopus oligosporus*. An important conclusion in both types of research is that fermentation also increases the protection of DNA against damage. In research by Bei et al. [69], oat flour was treated with the fungus *Monascus anka.* The results showed that the fermentation process changed the content of free and bound phenols compared to the non-fermented sample. The samples were extracted in three different ways depending on the type of phenolic fraction (free, conjugated, and bound). Generally, liquid-liquid extraction was used. HPLC with DAD was used to analyze phenolic fraction. Fermentation can also be an effective tool for the production of ellagic acid. It is the main compound found on the pomegranate husk after fermentation in the presence of *Aspergillus niger*. This approach was described by Sepúlveda [85]. The fungus *Aspergillus niger* was also used in research by Torres-León et al. [81] which showed that the fermentation of mango seeds with its participation improved nutraceutical properties and antioxidant activity. Rice fermentation assisted by *Monascus purpureus* in research by Pengnoi [70] confirmed an increase in monacolin K, citrinin, red pigments, and antioxidant properties. Additionally, it confirmed the strong correlation between the number of bioactive compounds and the variety of rice. A soybean by-product was treated with *S. cerevisiae* in research by Queiroz Santos [71] and a significant improvement in the physicochemical quality parameters was noted, as well as an increase in the content of total phenols, protein, and an improvement in antioxidant properties. The same conclusions were presented in the two articles by Sandhu et al. [67,77] where wheat and barley grains were fermented in the presence of the *Aspergillus awamorinakazawa* fungus strain.

## 4. The Concept of Enzymes and Enzymatic Extraction

Enzymes have been used for hundreds of years and their use today is almost limitless. The historical uses of enzymes in the production of beer, wine, cheese, and bread are elegant examples of the industrial exploitation of the power and selectivity of enzymes [87].

Enzymes are mostly macromolecular protein structures that accelerate chemical reactions by lowering their activation energy [88]. These biocatalysts bind to a wide range of molecules and arrange them in an optimal spatial configuration, allowing for effective breaking and forming of chemical bonds. The essence of catalytic proteins is to lower the activation energy and stabilize the transition states of reactions, which are the forms with the highest energy level [89].

Most of the enzymes known so far are proteins composed of chains from several dozen to several thousand amino acids [90,91]. These chains fold up to form a complex three-dimensional spatial structure. They can assume both secondary, tertiary, and quaternary structures [92]. There are places in the biocatalyst molecule where the binding of cofactors takes place—additional, non-protein components enabling the achievement of full catalytic activity. There are intracellular and extracellular enzymes which are catalysts of biological systems characterized by excellent properties due to their selectivity, specificity, and high activity. They make it possible to carry out even the most complex processes [93].

The most important influence on the catalytic properties of the enzyme is the spatial arrangement of amino acids that form the active center where the reaction takes place. High catalytic activity and specificity of enzymes and their regio- and chemoselectivity are also related to the shape and exposure of active centers of biocatalysts. These features make the biocatalyst capable of binding and reacting only with substrate molecules with a strictly defined arrangement of atoms and a defined chemical structure [94].

Enzymes are essential not only for the proper functioning of living organisms, but due to their unique properties and the ability to significantly increase the reaction rate, they have been used as effective catalysts in many technological processes [95].

Depending on the type of catalyzed reaction, enzymes are divided into six classes: oxidoreductases, transferases, hydrolases, lyases, isomerases, and ligases. Based on their specific catalytic properties, the enzyme can act on a specific substrate [94]. The division of enzymes into catalytic groups is presented in Table 2.

Plant cell walls are composed of about 90% polysaccharides and proteins constitute only 2–10% of the dry mass of the walls. Based on the existing models, it is suggested that the walls are a complex of interpenetrating networks connected by a multitude of covalent, hydrogen, and ionic bonds as well as hydrophobic interaction [102]. The main structural element is a cellulose-hemicellulose network, immersed in an amorphous matrix formed by a pectin network, enriched with proteins and phenolic compounds.

The synthesis and storage of plant bioactive substances are multi-stage processes—they take place both in the cytoplasm of plant cells and in the area of cell walls. Some bioactive compounds are bound in plant matrices by chemical bonds and are difficult to isolate through routine extraction using solvents. Bioactive compounds are localized intra- and extracellularly, some are bound by weak interactions, e.g., hydrophilic interactions or hydrogen bonds, with elements of cell walls, e.g., pectins, cellulose, and hemicellulose [103].

**Figure 8:**
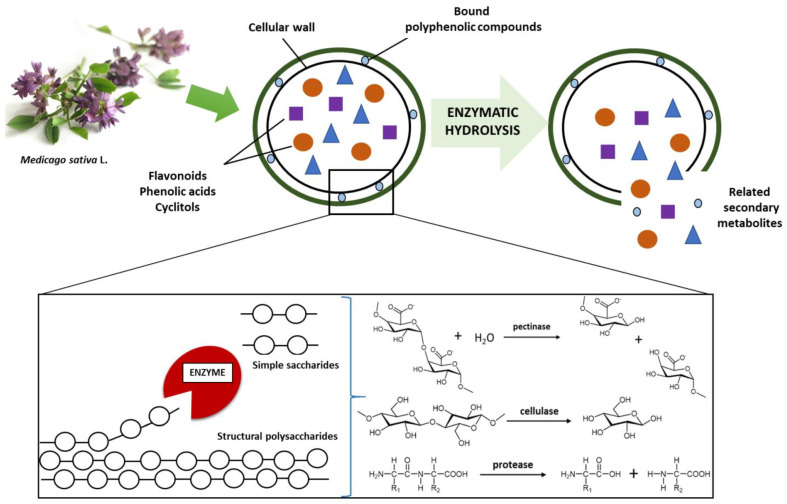
Plant cell wall degradation by enzyme.

Enzymes are ideal catalysts to aid the extraction, modification, or synthesis of complex bioactive compounds of natural origin. Enzyme-assisted extraction relies on the inherent ability of enzymes to catalyze reactions of exceptional specificity and selectivity, as well as the ability to function under mild processing conditions. The method also offers the possibility of greener chemistry as pressure is increasing on the food industry and pharmaceutical companies to identify cleaner extraction routes for new compounds. Enzymes have the ability to degrade or destroy cell walls and cell membranes, thus enabling better release and more efficient extraction of bioactive substances (Figure 8) [95,100].

The mechanism that determines the efficiency of extraction is the mass transport of the extracted substance from the inside of the plant material and then from the contact surface of the phases to the solvent. The key role in the extraction process is played by the resistance to mass transfer related to the structure of the raw material and the specific location of the extracted compounds [104,105]. The efficiency of the process is influenced by various factors: the type and catalytic properties of the enzyme, the composition and concentration of the reaction mixture, the particle size of plant materials, and the hydrolysis time [106].

The enzymatic pre-treatment techniques depend primarily on the ability of enzymes to hydrolyze components of the cell wall like cellulose, pectins, and hemicellulose. Degradation of these polymers disrupts the structural complexity of the cell wall and improves the extraction efficiency due to better mass transfer, particle size reduction, increased contact surface and enhanced solvent distribution. Moreover, enzymatic hydrolysis of the plant material results in the reduction of solvent consumption and extraction time [107,108,109]. However, the major disadvantage of this method is the necessity to select the conditions of enzymatic reactions.

The effectiveness of enzymatic hydrolysis depends on the type of enzymes and the selection of optimal conditions like pH, temperature, or ionic strength. For example, pre-treatment with cellulase was very effective in the extraction of phenolic compounds from grape marc. Cellulase and beta-glucosidase, used separately, significantly increase the efficiency of the isolation of polyphenolics from guava leaves. Enzymes can have a synergistic effect; combined pectinase and cellulase activity were very effective in extracting grape juice with high content of polyphenolic compounds from fox vine (*Vitis labrusca* L.) [110,111,112].

**Figure 9:**
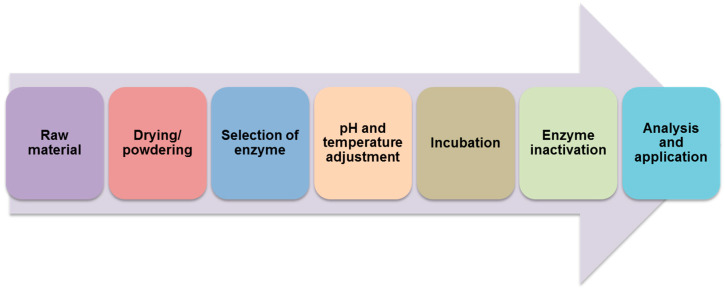
The steps of the enzyme-assisted extraction process.

The factors that influence the reaction rate are temperature, substrate and enzyme concentration, inhibitors, and pH. As the temperature rises, the activity of enzymes increases, to a certain point, after which it decreases [94]. As an example, to obtain an efficient enzymatic extraction for z-ligustilide from *Angelica sinensis*, factors influencing cellulase activity were investigated. It turned out that a pH below 5 and above 9 inhibited the enzymatic activity. The optimum pH was in the range of 7–8. The optimal temperature was 40 °C. The lower and higher temperatures were not appropriate for the enzyme activity [113]. Experiments with enzyme-assisted extraction are currently underway to determine the appropriate factors that will enable the isolation of the expected component (Figure 9).

An interesting example for the pre-treatment of plant material is the optimization of conditions of hydrolysis with the Box-Behnken design. In order to find the optimal conditions for hydrolysis, 27 experiments have been conducted with the use of four independent variables, i.e., pH, enzyme concentration, time, and temperature of the reaction. HPLC-MS/MS analysis showed that in the extracts obtained from the material previously subjected to enzymatic hydrolysis under optimal conditions, the total content of phenolic compounds obtained was about 2-fold higher compared to the control and 3.4-fold higher compared to the maceration extracts. It has also been shown that the initial treatment of plant material with an enzyme preparation commonly used in the agricultural industry significantly increased the efficiency of the extraction of bioactive substances. The use of enzyme-assisted supercritical fluid extraction in various industries to isolate bioactive compounds from plant material is economically effective and represents an advance in modern technological processes [12,114].

## 5. Conclusions

The pre-treatment of plant material is crucial for the extraction of bioactive compounds. On the laboratory scale, a wide range of methods and protocols are described in the literature. They involve drying, freeze-drying, convection drying, microwave vacuum drying, enzymatic processes, and fermentation. The selection of an extraction method should be based on the type of plant material and isolated compounds as well as prevent their degradation and the development of unfavorable microorganisms. Each selected method will affect the structure of plant material particles differently and subsequently alter the level of released bioactive compounds. However, an important challenge is the introduction of the described new, non-conventional methods of pre-treatment of plant material, such as freeze-drying, microwave vacuum drying, enzymatic processes, and fermentation, into the extraction processes on an industrial scale, which can significantly improve the efficiency of extraction of biologically active compounds. Therefore, further studies on the transfer of the mentioned methods into the industry, taking into account economical aspects, are still required.

## Figures and Tables

**Table 1:** The use of SSF in the production of bioactive compounds.

Natural Product	Bioactive Compound	Microorganism Used for Fermentation	Content of Bioactive Compounds in Fermented Samples	Reference
Barley grains	Polyphenols Flavonoids	*Aspergillus awamorinakazawa*	3786 ± 24–4954 ± 21 μg GAE/g (TPC) 2141 ± 16–2389 ± 15 μg CE/g (TFC)	[67]
Mung Beans	Polyphenols	*Cordyceps militaris SN-18*	5679.52 ± 57.29 μg GAE/g DW	[68]
Oats	Polyphenols	*Monascus anka*	355.07 ± 27.40 mg/kg (rutin)	[69]
Purple Rice	Antioxidant red pigments	*Monascus purpureus CMU002U*	388.25 OD/g of DW	[70]
Soybean okara	Polyphenols Isoflavones	*Saccharomyces cerevisiae*	116 mg GAE/10 g to 123 mg GAE/10 g	[71]
	Polyphenols	*Saccharomyces cerevisiae,* *Hansenula sp*	150 mg GA/100 g DW	[72]
Soybean	Vitamin K	*Bacillus subtilis NCIM 2708*	39.039 μg/g	[73]
	Polyphenols Isoflavones	*Rhizopus oligosporus RT-3*	3348.26 ± 39.44 to 7768.40 ± 171.27 mg GAE/g DW	[74]
		*Tricholoma matsutake*	1559.04 μg/g (isoflavones)	[75]
Wheat bran	Ferulic acid	*Aspergillus niger*	358.72 μg/g	[76]
Wheat grains	Polyphenols Flavonoids	*Aspergillus awamorinakazawa*	977–3598 μg GAE/g (TPC) 83–359 μg CE/g (TFC)	[77]
Apple Pomace	Polyphenols	*Phanerocheate chrysosporium*	4.6 to 16.12 mg GAE/g DW	[78]
Fig by-products		*Aspergillus niger HT4*	10.84 ± 0.39 mg of GAE/g DW	[79]
Garden cress seeds		*Trichoderma reesei*	3600 mg GAE/100 g DW	[80]
Mexican mango seed		*Aspergillus niger GH1*	3288 mg GAE/100 g (polyphenols)	[81]
Pineapple and guava fruit		*Rhizopus oligosporus*	from 14,691.5 ± 972.6 to 28,114.9 ± 1869.9 μg/g DW	[82]
Pineapple by-products		*Kluyveromyces marxianus NRRL Y-* *8281*	120 mg GA/100 g DW	[83]
Plum pomace	Polyphenols Flavonoids	*Aspergillus niger* *Rhizopus oligosporus*	119.75 ± 3.90 mg/100 g DW (flavonols) 59.58 ± 2.05 mg/100 g DW (cinnamic acids)	[84]
Pomegranate husk	Polyphenols	*Aspergillus niger*	47 mg/g	[85]
White grape pomace	Carotenoids γ-linolenic acid	*Actinomucor elegans* *Umbelopsis isabelline*	113.94 ± 3.42 mg/kg DW (phenolic acid) 343.95 ± 6.88 mg/kg DW (flavanols)	[86]

**Table 2:** Division of enzymes into catalytic groups along with the function performed by each of them and selected examples of enzymes.

Group Number	Group Name	The Type of Catalyzed Reaction	Exemplary Enzymes	Reference
I	oxidoreductases	Catalysis of oxidation and reduction reactions; transport of protons and electrons between reductant and oxidant molecules.	● dehydrogenase	[96]
II	transferases	Transferring the selected functional group from the donor molecule to the acceptor molecule.	● kinases	[97]
III	hydrolases	Catalysis of hydrolysis processes—the breakdown of chemical bonds with the participation of a water molecule.	● lipases	[98]
IV	lyases	Cleavage of functional groups from the substrate molecule and breakdown of the chemical bond by means other than hydrolysis or oxidation.	● aldolases	[99]
V	isomerases	Converting one isomeric form of a given compound to another.	● *cis-trans* isomerases	[100]
VI	ligases	Generation of new compounds by creating a chemical bond between two independent molecules.	● synthetases	[101]

## References

[1] Romanik G., Gilgenast E., Przyjazny A., Kamiński M. (2007). Techniques of preparing plant material for chromatographic separation and analysis. J. Biochem. Biophys. Methods.

[2] Moldoveanu S., David V. (2015). The role of sample preparation. Modern Sample Preparation for Chromatography.

[3] Sagandykova G.N., Pomastowski P., Buszewski B. (2020). Multi-instrumental approach to unravel molecular mechanisms of natural bioactive compounds: Case studies for flavonoids. TrAC.

[4] Brusottia G., Cesaria I., Dentamaroa A., Caccialanzaa G., Massolini G. (2014). Isolation and characterization of bioactive compounds from plant resources: The role of analysis in the ethnopharmacological approach. J. Pharm. Biomed. Anal..

[5] Rafińska K., Pomastowski P., Rudnicka J., Krakowska A., Maruška A., Narkute M., Buszewski B. (2019). Effect of solvent and extraction technique on composition and biological activity of *Lepidium sativum* extracts. Food Chem..

[6] Buszewski B., Szultka M. (2012). Past, Present, and Future of Solid Phase Extraction: A Review. Crit. Rev. Anal. Chem..

[7] Heras-Ramirez M.E., Quintero-Ramos A., Camacho-Davila A.A., Barnard J., Talamas-Abbud R., Torres-Munoz J.V., Salas-Muñoz E. (2012). Effect of blanching and drying temperature on polyphenolic compound stability and antioxidant capacity of apple pomace. Food Bioprocess Technol..

[8] Renard C.M.G.C., Watrelot A.A., Le Bourvellec C. (2017). Interactions between polyphenols and polysaccharides: Mechanisms and consequences in food processing and digestion. Trends Food Sci. Technol..

[9] Attokaran M. (2017). Preparation of plant material for extraction. Natural Food Flavors and Colorants.

[10] Zygmunt B., Namiesnik J. (2003). Preparation of samples of plant material for chromatographic analysis. J. Chromatogr. Sci..

[11] Jalan P., Zahir S., Pal T.K., Sengupta A., Biswas S., Bar S. (2019). Methods for preparing medicinal plant extracts: A review. Int. J. Curr. Res..

[12] Krakowska A., Rafińska K., Walczak J., Buszewski B. (2018). Enzyme-assisted optimized supercritical fluid extraction to improve *Medicago sativa* polyphenolics isolation. Ind. Crops Prod..

[13] Tiwari R.K.S., Chandravanshi S.S., Ojha B.M. (2005). Efficacy of extracts of medicinal plant species on growth of *Sclerotium rolfsii* root rot in tomato. Indian J. Mycol. Plant Pathol..

[14] Stępień B., Jałoszyński K., Szarycz M., Surma M., Pasławska M., Peroń S., Kamiński E., Stopa R., Jaźwiec B., Drożdż W. (2008). The impact of convection drying on selected mechanical and rheological properties of parsley root. Inżynieria Rol..

[15] Lewicki P.P. (2006). Design of hot air drying for better foods. Trends Food Sci. Technol..

[16] Roshanak S., Rahimmalek M., Goli S.A. (2016). Evaluation of seven different drying treatments in respect to total flavonoid, phenolic, vitamin C content, chlorophyll, antioxidant activity and color of green tea (*Camellia sinensis* or *C. assamica*) leaves. J. Food Sci. Technol..

[17] Thamkaew G., Sjöholm I., Galindo F.G. (2021). A review of drying methods for improving the quality of dried herbs. Crit. Rev. Food Sci. Nutr..

[18] Periche A., Castelló M.L., Heredia A., Escriche I. (2015). Influence of drying method on steviol glycosides and antioxidants in *Stevia rebaudiana* leaves. Food Chem..

[19] Nowak D., Syta M. (2009). Identification of the impact of grinding degree, pretreatment and drying method on content of betalaine dyes in dried beet material. Inżynieria Rol..

[20] Golisz E., Jaros M., Kalicka M. (2013). Analysis of convectional drying process of peach. Tech. Sci..

[21] Fernando W.J.N., Low H.C., Ahmad A.L. (2011). Dependence of the effective diffusion coefficient of moisture with thickness and temperature in convective drying of sliced materials. A study on slices of banana, cassava and pumpkin. J. Food Eng..

[22] Akbudak N., Akbudak B. (2013). Effect of vacuum, microwave, and convective drying on selected parsley quality. Int. J. Food Prop..

[23] Guiné R.P.F., Pinho S., Barroca M.J. (2011). Study of the convective drying of pumpkin (*Cucurbita maxima*). Food Bioprod. Process..

[24] Mrad N.D., Boudhrioua N., Kechaou N., Courtois F., Bonazzi C. (2012). Influence of air drying temperature on kinetics, physicochemical properties, total phenolic content and ascorbic acid of pears. Food Bioprod. Process..

[25] Lemus-Mondaca R., Ah-Hen K., Vega-Gálvez A., Honores K., Moraga N. (2016). *Stevia rebaudiana* leaves: Effect of drying process temperature on bioactive components, antioxidant capacity and natural sweeteners. Plant Foods Hum. Nutr..

[26] Gumusay O.A., Borazan A.A., Ercal N., Demirkol O. (2015). Drying effects on the antioxidant properties of tomatoes and ginger. Food Chem..

[27] Leng L.Y., Nadzri N., Shaari A.R., Norawanis A.R., Khor C.Y. (2017). Antioxidant capacity and total phenolic content of fresh, oven-dried and stir-fried tamarind leaves. Curr. Res. Nutr. Food Sci..

[28] Vongsak B., Sithisarn P., Mangmool S., Thongpraditchote S., Wongkrajang Y. (2013). Maximizing total phenolics, total flavonoids contents and antioxidant activity of *Moringa oleifera* leaf extract by the appropriate extraction method. Ind. Crops Prod..

[29] Shahhoseini R., Estaji A., Hosseini N., Ghorbanpour M., Omidbaigi R. (2013). The effect of different drying methods on the content and chemical composition of essential oil of lemon verbena (*Lippia citriodora*). J. Essent. Oil Bear. Plants..

[30] Khangholi S., Rezaeizadeh A. (2008). Effect of drying temperature on essential oil content and composition of sweet wormwood (*Artemisia annua*) growing wild in Iran. Pak. J. Biol. Sci..

[31] Sellami I.H., Wannes W.A., Bettaieb I., Berrima S., Chahed T., Marzouk B., Limam F. (2011). Qualitative and quantitative changes in the essential oil of *Laurus nobilis* L. leaves as affected by different drying methods. Food Chem..

[32] Gião M., Pereira C., Fonseca S., Pintado M., Malcata X. (2009). Effect of particle size upon the extent of extraction of antioxidant power from the plants *Agrimonia eupatoria*, *Salvia* sp. and *Satureja montana*. Food Chem..

[33] Louli V., Folas G., Voutsas E., Magoulas K. (2004). Extraction of parsley seed oil by supercritical CO_2_. J. Supercrit. Fluid..

[34] Calín-Sánchez Á., Lipan L., Cano-Lamadrid M., Kharaghani A., Masztalerz K., Carbonell-Barrachina Á.A., Figiel A. (2020). Comparison of Traditional and Novel Drying Techniques and Its Effect on Quality of Fruits, Vegetables and Aromatic Herbs. Foods.

[35] Kunal A.G., Mallinath H., Deepak B., Pallavi S.N. (2015). Lyophilization/freeze drying—A review. World J. Pharm. Res..

[36] Deepak B., Iqbal Z. (2015). Lyophilization—Process and optimization for pharmaceuticals. Int. J. Drug Regul. Aff..

[37] Kumar P. (2019). Lyophilization: An important formulation technique. Int. J. Res. Granthaalayah.

[38] Rezvankhah A., Emam-Djomeh Z., Askari G. (2020). Encapsulation and delivery of bioactive compounds using spray and freeze-drying techniques: A review. Dry. Technol..

[39] Oprica L., Antohe R.G., Verdes A., Grigore M.N. (2019). Effect of freeze-drying and oven-drying methods on flavonoids content in two romanian grape varieties. Rev. Chim..

[40] Elshaafi I.M., Musa K.H., Abdullah Sani N. (2020). Effect of oven and freeze drying on antioxidant activity, total phenolic and total flavonoid contents of fig (*Ficus carica* L.) leaves. Food Res..

[41] Jiang G.H., Lee K.C., Ameer K., Eun J.B. (2019). Comparison of freeze-drying and hot air-drying on Asian pear (*Pyrus pyrifolia Nakai* ‘Niitaka’) powder: Changes in bioaccessibility, antioxidant activity, and bioactive and volatile compounds. J. Food Sci. Technol..

[42] Pérez-Gregorio M.R., Regueiro J., González-Barreiro C., Rial-Otero R., Simal-Gándara J. (2011). Changes in antioxidant flavonoids during freeze-drying of red onions and subsequent storage. Food Control..

[43] Sun Y., Shen Y., Liu D., Ye X. (2015). Effects of drying methods on phytochemical compounds and antioxidant activity of physiologically dropped un-matured citrus fruits. LWT—Food Sci. Technol..

[44] Papoutsis K., Pristijono P., Golding J.B., Stathopoulos C.E., Bowyer M.C., Scarlett C.J., Vuong Q.V. (2017). Effect of vacuum-drying, hot air-drying and freeze-drying on polyphenols and antioxidant capacity of lemon (*Citrus limon*) pomace aqueous extracts. Int. J. Food Sci. Technol..

[45] Cui Z.W., Xu S.Y., Sun D.W. (2004). Microwave–vacuum drying kinetics of carrot slices. J. Food Eng..

[46] Kelen Á., Ress S., Nagy T., Pallai E., Pintye-Hódi K. (2006). Mapping of temperature distribution in pharmaceutical microwave vacuum drying. Powder Technol..

[47] Szarycz M., Kamiński E., Jałoszyński K., Szponarska A. (2003). Analiza mikrofalowego suszenia pietruszki w warunkach obniżonego ciśnienia. Część I. Kinetyka suszenia pietruszki nieblanszowanej i blanszowanej. Acta Sci. Pol. Tech. Agrar..

[48] Stępień B. (2007). Impact of the drying method on the process of carrot cutting. Acta Agroph..

[49] Ferenczi S., Czukor B., Cserhalmi Z. (2014). Evaluation of microwave vacuum drying combined with hot-air drying and compared with freeze- and hot-air drying by the quality of the dried apple product. Per. Pol. Chem. Eng..

[50] Berteli M.N., Rodier E., Marsaioli A. (2009). Study of the microwave vacuum drying process for a granulated product. Braz. J. Chem. Eng..

[51] Li Y., Xu S.Y., Sun D.W. (2007). Preparation of garlic powder with high allicin content by using combined microwave–vacuum and vacuum drying as well as microencapsulation. J. Food Eng..

[52] Therdthai N., Zhou W. (2009). Characterization of microwave vacuum drying and hot air drying of mint leaves (*Mentha cordifolia Opiz ex Fresen*). J. Food Eng..

[53] Giri S.K., Prasad S. (2007). Drying kinetics and rehydration characteristics of microwave-vacuum and convective hot-air dried mushrooms. J. Food Eng..

[54] Motavali A., Najafi G.H., Abbasi S., Minaei S., Ghaderi A. (2011). Microwave–vacuum drying of sour cherry: Comparison of mathematical models and artificial neural networks. J. Food Sci. Technol..

[55] Manzocco L., Calligaris S., Mastrocola D., Nicoli M., Larici C. (2001). Review of non-enzymatic browning and antioxidant capacity in processed foods. Trends Food Sci. Technol..

[56] Karata F., Kamışlı F. (2007). Variations of vitamins (A, C and E) and MDA in apricots dried in IR and microwave. J. Food Eng..

[57] Lin T.M., Durance T.D., Scaman C.H. (1998). Characterization of vacuum microwave, air and freeze dried carrot slices. Food Res. Int..

[58] Alibas I.O., Akbudak B., Akbudak N. (2007). Microwave drying characteristics of spinach. J. Food Eng..

[59] Alibas I. (2010). Determination of drying parameters, ascorbic acid contents and color characteristics of nettle leaves during microwave-, air- and combined microwave-air drying. J. Food Process. Eng..

[60] Wojdyło A., Figiel A., Oszmiański J. (2009). Effect of drying methods with the application of vacuum microwaves on the bioactive compounds, color, and antioxidant activity of strawberry fruits. J. Agric. Food Chem..

[61] Zielińska M., Michalska A. (2016). Microwave-assisted drying of blueberry (*Vaccinium corymbosum* L.) fruits: Drying kinetics, polyphenols, anthocyanins, antioxidant capacity, colour and texture. Food Chem..

[62] Ali A., Oon C.C., Chua B.L., Figiel A., Chong C.H., Wojdylo A., Turkiewicz I.P., Szumny A., Łyczko J. (2020). Volatile and polyphenol composition, anti-oxidant, anti-diabetic and antiaging properties, and drying kinetics as affected by convective and hybrid vacuum microwave drying of *Rosmarinus officinalis* L.. Ind. Crops Prod..

[63] Netsanet S.T., Smithers G. (2016). Food fermentation. Elsevier Food Science Reference Module.

[64] Cano y Postigo L.O., Jacobo-Velazquez D.A., Guajardo-Flores D., Garcia Amezquita L.E., García-Cayuela T. (2021). Solid-state fermentation for enhancing the nutraceutical content of agrifood by-products: Recent advances and its industrial feasibility. Food Biosci..

[65] Subramaniyam R., Vimala R. (2012). Solid state and submerged fermentation for the production of bioactive substances: A comparative study. IJSN.

[66] Ramos O.S., Malcata F.X. (2011). Food-Grade Enzymes. Comprehensive Biotechnology.

[67] Sandhu K.S., Punia S. (2017). Enhancement of bioactive compounds in barley cultivars by solid substrate fermentation. J. Food Meas. Charact..

[68] Xiao Y., Zhang Q., Miao J., Rui X., Li T., Dong M. (2015). Antioxidant activity and DNA damage protection of mung beans processed by solid state fermentation with *Cordyceps militaris* SN-18. IFSET.

[69] Bei Q., Liu Y., Wang L., Chen G., Wu Z. (2017). Improving free, conjugated, and bound phenolic fractions in fermented oats (*Avena sativa* L.) with *Monascus anka* and their antioxidant activity. J. Funct. Foods.

[70] PengnOi P., Mahawan R., KhanOngnuCh C., LuMyOng S. (2017). Antioxidant properties and production of monacolin K, citrinin, and red pigments during solid state fermentation of purple rice (*Oryzae sativa*) varieties by Monascus purpureus. Czech J. Food Sci..

[71] Queiroz Santos V.A., Nascimento C.G., Schmidt C.A.P., Mantovani D., Dekker R.F.H., da Cunha M.A.A. (2018). Solid-state fermentation of soybean okara: Isoflavones biotransformation, antioxidant activity and enhancement of nutritional quality. LWT—Food Sci. Technol..

[72] Shi H., Zhang M., Wang W., Devahastin S. (2020). Solid-state fermentation with probiotics and mixed yeast on properties of okara. Food Biosci..

[73] Singh R., Puri A., Panda B.P. (2015). Development of menaquinone-7 enriched nutraceutical: Inside into medium engineering and process modeling. J. Food Sci. Technol..

[74] Xiao Y., Fan J., Chen Y., Rui X., Zhang Q., Dong M. (2016). Enhanced total phenolic and isoflavone aglycone content, antioxidant activity and DNA damage protection of soybeans processed by solid state fermentation with *Rhizopus oligosporus* RT-3. RSC Adv..

[75] Lee J.H., Hwang C.E., Son K.S., Cho K.M. (2019). Comparisons of nutritional constituents in soybeans during solid state fermentation times and screening for their glucosidase enzymes and antioxidant properties. Food Chem..

[76] Yin Z.N., Wu W.J., Sun C.Z., Liu H.F., Chen W.B., Zhan Q.P., Lei Z.G., Xin X., Ma J.J., Yao K. (2019). Antioxidant and anti-inflammatory capacity of ferulic acid released from wheat bran by solid-state fermentation of *Aspergillus niger*. Biomed. Environ. Sci..

[77] Sandhu K.S., Punia S., Kaur M. (2016). Effect of duration of solid state fermentation by *Aspergillus awamorinakazawa* on antioxidant properties of wheat cultivars. LWT—Food Sci. Technol..

[78] Ajila C.M., Brar S.K., Verma M., Tyagi R.D., Valéro J.R. (2011). Solid-state fermentation of apple pomace using Phanerocheate chrysosporium—Liberation and extraction of phenolic antioxidants. Food Chem..

[79] Buenrostro-Figueroa J.J., Velázquez M., Flores-Ortega O., Ascacio-Valdés J.A., Huerta-Ochoa S., Aguilar C.N., Prado-Barragán L.A. (2017). Solid state fermentation of fig (*Ficus carica* L.) by-products using fungi to obtain phenolic compounds with antioxidant activity and qualitative evaluation of phenolics obtained. Process. Biochem..

[80] Abdel-Aty A.M., Bassuiny R.I., Barakat A.Z., Mohamed S.A. (2019). Upgrading the phenolic content, antioxidant and antimicrobial activities of garden cress seeds using solid-state fermentation by Trichoderma reesei. J. Appl. Microbiol..

[81] Torres-Léon C., Ramírez-Guzmán N., Ascacio-Valdés J., Serna-Cock L., dos Santos Correia M.T., Contreras-Esquivel J.C., Aguilar C.N. (2019). Solid-state fermentation with *Aspergillus Niger* to enhance the phenolic contents and antioxidative activity of Mexican mango seed: A promising source of natural antioxidants. LWT—Food Sci. Technol..

[82] Sousa B.A., Correia R.T.P. (2012). Phenolic content, antioxidant activity and antiamylolytic activity of extracts obtained from bioprocessed pineapple and guava wastes. Braz. J. Chem. Eng..

[83] Rashad M.M., Mahmoud A.E., Ali M.M., Nooman M.U., Al-Kashef A.S. (2015). Antioxidant and anticancer agents produced from pineapple waste by solid state fermentation. Int. J. Toxicol. Pharmacol. Res..

[84] Dulf F.V., Vodnar D.C., Socaciu C. (2016). Effects of solid-state fermentation with two filamentous fungi on the total phenolic contents, flavonoids, antioxidant activities and lipid fractions of plum fruit (*Prunus domestica* L.) by-products. Food Chem..

[85] Sepúlveda L., Wong-Paz J.E., Buenrostro-Figueroa J., Ascacio-Valdés J.A., Aguilera-Carbó A., Aguilar C.N. (2018). Solid state fermentation of pomegranate husk: Recovery of ellagic acid by SEC and identification of ellagitannins by HPLC/ESI/MS. Food Biosci..

[86] Dulf F.V., Vodnar D.C., Toşa M.I., Dulf E.-H. (2020). Simultaneous enrichment of grape pomace with γ-linolenic acid and carotenoids by solid-state fermentation with Zygomycetes fungi and antioxidant potential of the bioprocessed substrates. Food Chem..

[87] Baby K.C., Ranganathan T.V. (2013). Enzyme-assisted extraction of bioingredients. Chem. Wkly..

[88] Fersht A. (1999). Structure and Mechanism in Protein Science: A Guide to Enzyme Catalysis and Protein Folding.

[89] Pelley J.W. (2012). Elsevier’s Integrated Review Biochemistry.

[90] Chen L.H., Kenyon G.L., Curtin F., Harayama S., Bembenek M.E., Hajipour G., Whitman C.P. (1992). 4-Oxalocrotonate tautomerase, an enzyme composed of 62 amino acid residues per monomer. J. Biol. Chem..

[91] Smith S. (1995). The animal fatty acid synthase: One gene, one polypeptide, seven enzymes. FASEB J..

[92] Sowbhagya H.B., Chitra V.N. (2010). Enzyme-assisted extraction of flavorings and colorants from plant materials. Crit. Rev. Food Sci. Nutr..

[93] Mateo C., Palomo J.M., Fernangez-Lorente G. (2007). Improvement of enzyme activity, stability and selectivity via immobilization techniques. Enzyme Microb. Technol..

[94] Rodwell V.W., Bender D.A., Botham K.M., Kennelly P.J., Weil A.P. (2018). Harper’s Illustrated Biochemistry.

[95] Koshland D.E. (1985). Application of a theory of enzyme specificity to protein synthesis. Proc. Natl. Acad. Sci. USA.

[96] Luo J., Meyer A.S., Mateiu R.V., Pinelo M. (2015). Cascade catalysis in membranes with enzyme immobilization for multienzymatic conversion of CO_2_ to methanol. New Biotechnol..

[97] Bleeker F.E., Lamba S., Zanon C., Molenaar R.J., Hulsebos T.J., Troost D., van Tilborg A.A., Vandertop W.P., Leenstra S., van Noorden C.J. (2014). Mutational profiling of kinases in glioblastoma. BMC Cancer.

[98] Schmid R.D., Verger R. (1998). Lipases: Interfacial enzymes with attractive applications. Angew. Chem. Int. Ed..

[99] Clapes P., Fessner W.D., Sprenger G.A., Samland A.K. (2010). Recent progress in stereoselective synthesis with aldolases. Curr. Opin. Chem. Biol..

[100] Bhosale S.H., Rao M.B., Deshpande V.V. (1996). Molecular and industrial aspects of glucose isomerase. Microbiol. Rev..

[101] Pascal J.M., Tsodikov O.V., Hura G.L., Song W., Cotner E.A., Classen S., Tomkinson A.E., Tainer J.A., Ellenberger T. (2006). A flexible interface between DNA ligase and PCNA supports conformational switching and efficient ligation of DNA. Mol. Cell.

[102] Somerville C., Bauer S., Brininstool G., Facette M., Flamann T., Milne J., Osborne E., Paredez A., Persson S., Raab T. (2004). Toward a systems approach to understanding plant cell walls. Science.

[103] Kutchan T.M. (2005). A role for intra- and intercellular translocation in natural product biosynthesis. Curr. Opin. Plant Biol..

[104] Mushtaq M., Sultana B., Bhatti H.N., Asghar M. (2015). RSM based optimized enzyme-assisted extraction of antioxidant phenolics from underutilized watermelon *(Citrullus lanatus Thunb.)* rind. J. Food Sci. Technol..

[105] Qin Y., Yuan Q., Zhang Y., Li J., Zhu X., Zhao L., Wen J., Liu J., Zhao L., Zhao J. (2018). Enzyme-assisted extraction optimization, characterization and antioxidant activity of polysaccharides from sea cucumber *Phyllophorus proteus*. Molecules.

[106] Azmir J., Zaidul I.S.M., Rahman M.M., Sharif K.M., Mohamed A., Sahena F., Jahurul M.H.A., Ghafoor K., Norulaini N.A.N., Omar A.K.M. (2013). Techniques for extraction of bioactive compounds from plant materials: A review. J. Food Eng..

[107] Fu Y.J., Liu W., Zu Y.G., Tong M.H., Li S.M., Yan M.M., Efferth T., Luo H. (2008). Enzyme assisted extraction of luteolin and apigenin from pigeonpea [*Cajanuscajan* (L.) *Millsp.*] leaves. Food Chem..

[108] Mushtaq M., Sultana B., Akram S., Anwar F., Adnan A., Rizvi S.S.H. (2017). Enzyme-assisted supercritical fluid extraction: An alternative and green technology for non-extractable polyphenols. Anal. Bioanal. Chem..

[109] Cho J.H., Saurabh B., Tae-Jin O., Jong H.J. (2013). Enzymatic extraction of pilocarpine from *Pilocarpus jaborandi*. Korean J. Microbiol. Biotechnol..

[110] Li B.B., Smith B., Hossain M.M. (2006). Extraction of phenolics from citrus peels: II. Enzyme assisted extraction method. Sep. Purif. Technol..

[111] Gómez-García R., Martínez-Ávila G.C.G., Aguilar C.N. (2012). Enzyme assisted extraction of antioxidative phenolics from grape (*Vitis vinifera* L.) residues. 3 Biotech.

[112] Wang L., Wu Y., Liu Y., Wu Z. (2017). Complex enzyme-assisted extraction releases antioxidative phenolic compositions from guava leaves. Molecules.

[113] Zhang X.-G., Lu Y., Wang W.-N., Liu Z.-Y., Liu J.-W., Chen X.-Q. (2017). A novel enzyme-assisted approach for efficient extraction of Z-ligustilide from *Angelica sinensis* plants. Sci. Rep..

[114] Krakowska-Sieprawska A., Rafińska K., Walczak-Skierska J., Kiełbasa A., Buszewski B. (2021). Promising green technology in obtaining functional plant preparations: Combined enzyme-assisted supercritical fluid extraction of flavonoids isolation from *Medicago sativa* leaves. Materials.

